# Mechanistic studies of *miR*-582-3p targeting of *PTPRCAP* affecting lung adenocarcinoma via the Wnt/β-catenin pathway

**DOI:** 10.3389/fonc.2025.1652176

**Published:** 2025-09-23

**Authors:** Yuting Yang, Song Zhao, Xiaoli Han, Pengfei Guo, Baoshan Zhao, Zongying Liang

**Affiliations:** ^1^ Department of Thoracic Surgery, Affiliated Hospital of Chengde Medical University, Chengde, Hebei, China; ^2^ Hebei Key Laboratory of Panvascular Disease, Chengde, Hebei, China

**Keywords:** miR-582-3p, PTPRCAP, lung adenocarcinoma, Wnt/β-catenin, signaling pathways

## Abstract

**Objective:**

To investigate the regulatory mechanism by which MicroRNA-582-3p (*miR*-582-3p) targets protein tyrosine phosphatase receptor type C-associated protein (*PTPRCAP*) and modulates Wnt/β-catenin signaling in lung adenocarcinoma pathogenesis.

**Methods:**

Bioinformatics analysis of TCGA data assessed *miR*-582-3p expression and its clinicopathological relevance in LUAD. *PTPRCAP* mRNA and protein levels were evaluated via RT-qPCR and immunohistochemistry. The *miR*-582-3p*-PTPRCAP* interaction was validated using TargetScan8.0 and dual-luciferase reporter assays. Functional assays (CCK-8, scratch, Transwell) determined the effects of *miR*-582-3p and *PTPRCAP* on LUAD cell proliferation, migration, and invasion. Western blotting analyzed Wnt/β-catenin pathway components (β-catenin, GSK3β, p-GSK3β).

**Results:**

*miR*-582-3p was significantly upregulated in LUAD tissues and cell lines (A549, H1299), correlating with advanced disease features. *PTPRCAP*, a predicted target of *miR*-582-3p, showed reduced expression in LUAD. Dual-luciferase assays confirmed *miR*-582-3p directly binds the *PTPRCAP* 3′-UTR (P < 0.05). Overexpressing *miR*-582-3p suppressed *PTPRCAP*, enhanced malignant phenotypes (P < 0.05), and activated Wnt/β-catenin signaling (increased β-catenin and p-GSK3β; decreased GSK3β). Conversely, *PTPRCAP* overexpression inhibited tumorigenic behaviors and Wnt pathway activity. Rescue experiments demonstrated that *PTPRCAP* restoration counteracted *miR*-582-3p–mediated oncogenic effects (P < 0.05).

**Conclusion:**

Our findings reveal a novel *miR*-582-3p/*PTPRCAP*/Wnt/β-catenin axis in LUAD progression, where *miR*-582-3p drives tumor growth by silencing *PTPRCAP* and activating Wnt signaling. These results highlight *miR*-582-3p as a potential therapeutic target and *PTPRCAP* as a tumor suppressor in LUAD, offering new insights for targeted intervention strategies.

## Introduction

Lung cancer remains the leading cause of cancer-related mortality worldwide, with lung adenocarcinoma (LUAD) accounting for approximately 40% of all lung cancer cases (1, 2). Owing to the absence of specific early symptoms, most patients are diagnosed at advanced stages, frequently accompanied by local invasion and distant metastasis, resulting in a 5-year overall survival rate below 20% (3). Advances in modern biomedical technologies and the integration of multidisciplinary approaches have opened new therapeutic avenues for LUAD; however, a deeper understanding of its molecular pathogenesis is still required to identify effective therapeutic targets (4).

Protein tyrosine phosphatase receptor type C-associated protein (*PTPRCAP*) belongs to the PTPR family and directly interacts with PTPRC, thereby stabilizing its expression. PTPRC encodes CD45, one of 18 PTP genes among 72 survival phosphatases (5). Mounting evidence demonstrates that *PTPRCAP* down-regulation promotes immune evasion in colorectal cancer (6) and associates with adverse clinicopathological features and poor prognosis in hepatocellular carcinoma (7, 8). Moreover, our recent single-cell RNA-seq study revealed that *PTPRCAP* is up-regulated in NK and B cells of patients with carbapenem-resistant Klebsiella pneumoniae (CRKP) pneumonia, suggesting its potential as an immune-status biomarker (9). While PTPRC has been reported to modulate epithelial–mesenchymal transition (EMT) via the Wnt signaling pathway in non-small-cell lung cancer (NSCLC) (10), the precise role of its binding partner *PTPRCAP* in LUAD remains largely unexplored.

MicroRNAs (*miR*NAs) are a class of small non-coding RNAs that post-transcriptionally regulate gene expression by binding to complementary sequences in target mRNAs (11). Aberrant expression of multiple *miR*NAs has been documented in NSCLC and is implicated in tumorigenesis and progression (12, 13). Among them, *miR*-582-3p has been shown to suppress prostate cancer bone metastasis by inhibiting the TGF-β pathway (14), to control proliferation and invasion in hepatocellular carcinoma (15), and to be sponged by lncRNA PRKCQ-AS1 in LUAD, thereby regulating downstream gene expression (16). In silico analysis using TargetScan predicts that *miR*-582-3p harbors potential binding sites within the 3′-UTR of *PTPRCAP*, suggesting the existence of a novel *miR*-582-3p/*PTPRCAP* regulatory axis.

The Wnt/β-catenin signaling cascade is a pivotal pathway driving tumor progression (17). Aberrant activation of this pathway has been described in breast (18), gastric (19), cervical (20), and lung cancers (21). Specifically, *miR*-1246 promotes NSCLC metastasis by targeting GSK-3β and activating Wnt/β-catenin signaling (21). Recent evidence further indicates that *miR*-582-3p can enhance Wnt/β-catenin pathway activity (22). We therefore hypothesize that *miR*-582-3p may promote LUAD progression by directly targeting *PTPRCAP* and concomitantly activating the Wnt/β-catenin pathway.

Based on the above background, this study integrates clinical specimens, functional assays, and animal models with bioinformatics and molecular biology to elucidate how *miR*-582-3p targets *PTPRCAP* and, via the Wnt/β-catenin pathway, drives LUAD progression, thereby providing new molecular insights.

## Materials and methods

### Data source

We obtained *miR*NA-seq data from 33 cancer types in the TCGA database (https://portal.gdc.cancer.gov/), including 521 primary lung adenocarcinoma (LUAD) tumor samples and 46 paired adjacent normal lung tissues. The raw sequencing data were processed using the BCGSC *miR*NA Profiling Pipeline and normalized to reads per million (RPM) mapped reads. All analyses were performed in R (version 4.2.1) without log transformation or batch correction to maintain data integrity. Corresponding clinical data were retrieved from the TCGA-LUAD dataset for integrated analysis.

### Tissue and cell

A total of 45 paired tumor and adjacent normal tissue specimens were obtained from lung adenocarcinoma patients undergoing surgical resection at the Department of Thoracic Surgery, Affiliated Hospital of Chengde Medical University. All specimens were immediately snap-frozen in liquid nitrogen following surgical resection and stored at -80 °C until subsequent experiments. Among them, 27 were female and 18 were male; 28 cases were ≥ 60 years old and 17 cases were < 60 years old, with an average age of (62.29 ± 7. 45) years old; 23 cases were in stage I and 22 cases were in stage II; 3 cases were poorly differentiated, 39 cases were moderately differentiated and 3 cases were well differentiated; Lymphatic metastasis in 6 cases. Inclusion criteria: 1. Patients with lung adenocarcinoma confirmed by pathology; 2. No anti-tumor treatment (radiotherapy, chemotherapy, immunotherapy, or anti-tumor ready-for-use traditional Chinese medicine treatment) has been performed before taking the specimen; 3 Patients who have never had any other malignant tumors. Exclusion criteria: 1. Patients with incomplete data and or other malignant tumors; 2. Patients who had undergone radiotherapy and chemotherapy, and other anti-tumor treatments, before surgery. Human lung adenocarcinoma cells A549, H1299, and normal lung epithelial cells BEAS-2B were derived from the central laboratory of Affiliated Hospital of Chengde Medical University. The study was approved by the hospital ethics committee, and informed consent was obtained from patients.

### Reagents and instruments

Serum and basal medium were purchased from Punosai Life Technology Co., Ltd.; *miR*-582-3p mimics (*miR*-582-3p mimics) and negative control (mimics NC) were purchased from Anhui Jinbiao Biotechnology Co., Ltd.; *PTPRCAP* overexpression plasmid was purchased from Nanjing Jingpusaier Biotechnology Co., Ltd.; Lipofectamine 3000 transfection reagent, *PTPRCAP* primer and GAPDH primer were purchased from Invitrogen, USA; Dual-Lucifarase Reporter Assay System was purchased from Promega Corporation, USA; *miR*-582-3p primer and U6 primer were purchased from Tiangen Biochemical Technology (Beijing) Co., Ltd.; Reverse transcription kit, real-time fluorescent polymerase chain reaction (RT-qPCR) kit and CCK-8 kit were purchased from Cisco Biotechnology Co., Ltd.; Matrigel was purchased from Biozellen Corporation, USA; *PTPRCAP* antibody was purchased from Wuhan Sanying Biotechnology Co., Ltd., China, and GAPDH antibody was purchased from Wuhan Sevier Biotechnology Co., Ltd.; β-catenin antibody was purchased from Huaan Biotechnology Co., Ltd.; GSK3β and p-GSK3β antibodies were purchased from Ebiwei Biotechnology Co., Ltd.; Goat anti-rabbit immunoglobulin G secondary antibody was purchased from Aibotek Biotechnology Co., Ltd.

### Cell culture, transfection, and grouping

BEAS-2B, A549, and H1299 cells were resuscitated and passaged in DMEM, RPMI-1640, and F12K medium, respectively, supplemented with 10% fetal bovine serum (FBS) and 1% penicillin-streptomycin, followed by incubation in a constant-temperature incubator. A549 and H1299 cells were seeded into 6-well plates, and upon reaching approximately 80% confluence, they were transfected using Lipofectamine 3000 transfection reagent. The cells were divided into the following groups: the miR-582-3p group, miR-NC group, OE group, Vector group, miR-582-3p + OE group, and miR-582-3p + Vector group. Specifically, the miR-582-3p group and miR-NC group were transfected with miR-582-3p mimics and mimics NC at a final concentration of 100 nM, respectively; the OE group and Vector group were transfected with the PTPRCAP overexpression plasmid and the corresponding control plasmid at 2500 ng per well, respectively; and the miR-582-3p + OE group and miR-582-3p + Vector group were co-transfected with the respective reagents. After transfection, cells were initially cultured in basal medium for 24 h, followed by replacement with complete medium containing 10% FBS for an additional 24 h before subsequent experiments.

### RNA extraction, reverse transcription, and real-time fluorescence quantitative polymerase reaction

The tissues were ground with a low-temperature tissue homogenizer, and the total RNA of tissues and cells was extracted with Trizol reagent, and the RNA was reverse transcribed into cDNA by a reverse transcription kit. The configuration of the RT-qPCR system and the reaction conditions were carried out in strict accordance with the instructions of the fluorescence quantification kit. The relative quantities were calculated using the 2^-ΔΔ^ct method. *PTPRCAP* forward primer sequence 5’-CAGGACACACAGACTATGACCACG-3’ ´; Reverse primer sequence 5’-GTCACTGTCTCTGGCTTCCTCA-3’. *GAPDH* forward primer sequence 5’-CGACCACTTTGACAAGCTCA-3’ ´, reverse primer sequence 5’-AGGGGTCTACATGGCAACTG-3’ ´. *miR*-582-3p forward primer sequence 5’-UCAGUGACAGUAGUUUGUCAAG-3’; Reverse primer sequence 5’-CCAGTGCAGGGTCCGAGGT-3’. *U6* forward primer sequence 5’-CTCGCTTCGGCAGCACA-3’; Reverse primer sequence 5’-AACGCTTCACGAATTTGCGT-3’.

### Immunohistochemistry

Tissue sections were dewaxed, repaired by microwave antigen retrieval, and incubated in 3% hydrogen peroxide blocking solution for 15 minutes. After the sections were cooled, 10% goat serum was blocked; Primary antibody (PTPRCAP 1: 500) was added dropwise at 4°C overnight; The next day, the primary antibody was rewarmed for 1h; Add secondary antibody dropwise and incubate at 37°C for 30 min; DBA color development, hematoxylin counterstaining, dehydration transparency, gum sealing, microscope observation results. The percentage score of positive cells was 0-4 (0 was 0%-5%, 1 was 5%-24%, 2 was 25%-49%, 3 was 50%-74%, and 4 was 75%-100%). The staining intensity score was 0 ~ 3 (0 was negative staining, 1 was weak staining, 2 was moderate staining, and 3 was strong staining). Immunoreactive Score (IRS) was calculated as IRS = PP*SI, where PP represents the score of the percentage of positive cells and SI is the level of staining intensity.IRS ≤ 2 was classified as low expression, and > 2 as high expression.

### Dual luciferase gene reporter assay

Potential interactions between *miR*-582-3p and *PTPRCAP*, along with the predicted binding sites, were identified using Target Scan. To experimentally validate this interaction, we constructed wild-type (*WT-PTPRCAP*) and mutant (*MUT-PTPRCAP*) luciferase reporter vectors containing the putative *miR*-582-3p binding sequence. A549 and H1299 cells were seeded in 6-well plates and co-transfected with either *miR*-582-3p mimics or mimics NC, along with the respective reporter vectors (*WT-PTPRCAP* or *MUT-PTPRCAP*). Following transfection, cells were cultured in basal medium for 24 h, followed by replacement with complete medium (10% FBS) for an additional 24 h. Luciferase activity was measured using a dual-luciferase reporter assay system, and the relative activity was determined by calculating the ratio of firefly luciferase to Renilla luciferase luminescence. This assay confirmed the regulatory effect of *miR*-582-3p on *PTPRCAP* expression.

### Western blot experiment

Total protein was extracted from each experimental group and quantified. Protein samples were separated by SDS-PAGE (160 V constant voltage) and transferred to methanol-activated PVDF membranes (400 mA constant current). After transfer, membranes were blocked with 5% skim milk in TBST for 2h at room temperature, followed by incubation with primary antibodies: anti-PTPRCAP (1:1000), anti-GAPDH (1:4000), anti-β-catenin (1:1000), anti-GSK3β (1:2000), and anti-p-GSK3β (1:4000) at 4 °C overnight. The next day, membranes were rewarmed for 1 h, washed with TBST (3×10 min), and incubated with HRP-conjugated secondary antibody (1:10000) for 1h. After final washes (3×10 min TBST), protein bands were visualized using the C300 imaging system. Band intensities were quantified using ImageJ software by calculating the ratio of target protein to GAPDH signal.

### CCK-8 assay to detect cell proliferation activity

The cell suspensions were counted and seeded into 96-well plates with 6 replicate wells in each group, and 100 μl of cell suspension containing 2500 cells was added to each well. After the cells were cultured for 0 h, 24 h, 48 h, 72 h, 10 μl of CCK-8 reagent was added to each well. After continued incubation in a 37°C incubator for 2 hours, the absorbance value (OD value) at 450 nm wavelength was measured with a microplate reader.

### Scratch healing experiment

A549 cells and H1299 cells were seeded in 6-well plates, with 3 double wells in each group. When the cell density reaches about 80%, transfection is carried out. After the cells are cultured until the bottom of the well is covered, the bottom of the vertical well plate is scratched with the tip of a 10 μl pipette. The floating cells were washed off with PBS and added to basal medium for culture. The scratched areas were photographed under an inverted microscope at 0 h and 24 h, respectively.

### Transwell cell migration and invasion experiment

Migration assay: Forty-eight hours post-transfection, cells (3×10^4^/well) in 1% FBS medium were seeded into the upper chamber, while the lower chamber contained 700 μl of 20% FBS medium. After 24 h incubation, non-migrated cells were removed by a cotton swab. Cells that migrated through the membrane were fixed with methanol, stained with 1% crystal violet, and quantified under an inverted microscope. Invasion assay: The upper chamber was pre-coated with Matrigel (Corning). Cells (5×10^4/^well) were seeded as described for the migration assay, with subsequent steps performed identically.

### Statistical analysis

The data were statistically analyzed and plotted using Graphpad Prism 10.0 software. The measurement data obeying the normal distribution is represented by; t-test was used for data comparison between the two groups, and One-way ANOVA or two-way ANOVA was used for data comparison between multiple groups; Wilcoxon rank sum test was used for data that did not obey the normal distribution. Count data are shown as [Example (%)] using a paired four-cell Table χ2 test. The difference was statistically significant with P < 0.05. (*P < 0.05, **P < 0.01, ***P < 0.001, ****P < 0.001).

## Results

### Expression of miR-582-3p and *PTPRCAP* in lung adenocarcinoma and their targeting relationship

**Figure 1:**
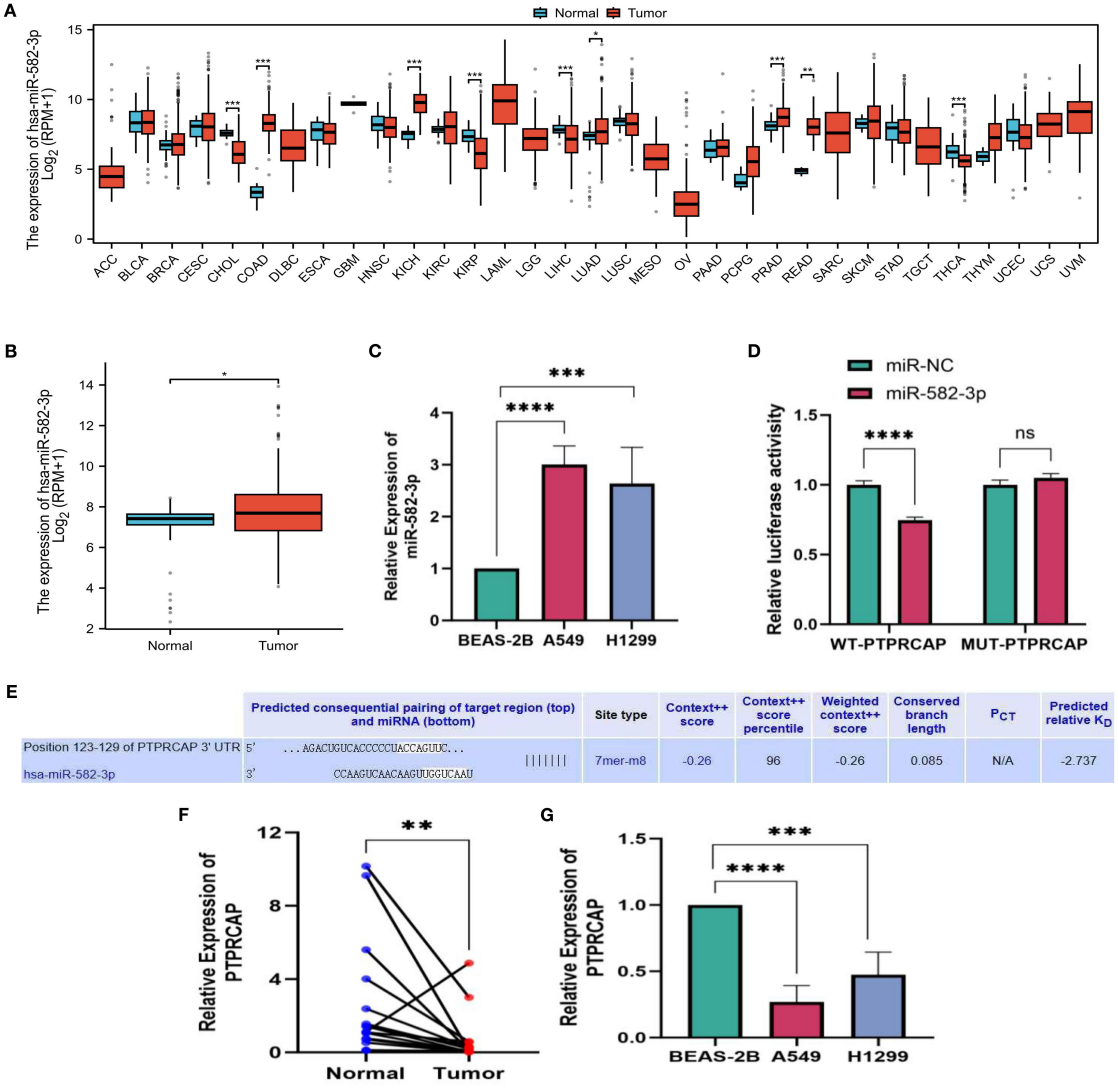
miR-582-3p, expression level of *PTPRCAP*. **(A)** Expression of miR-582-3p in 33 cancers in TCGA database. **(B)** Differential expression of miR-582-3p in TCGA-LUAD data. **(C)** Expression of miR-582-3p in BEAS-2B, A549 and H1299 cells **(D)** Luciferase reporter gene experiment of miR-582-3p target gene *PTPRCAP*. **(E)** Target Scan bioinformatics database predicts binding sites for miR-582-3p and *PTPRCAP*. **(F)** Relative expression of *PTPRCAP* in cancer tissues and adjacent tissues of 18 patients with lung adenocarcinoma **(G)** Relative expression of *PTPRCAP* mRNA in BEAS-2B, A549 and H1299 cells. *P < 0.05, **P < 0.01, ***P < 0.001, ****P < 0.001.

We downloaded data from the TCGA database and analyzed the expression of *miR*-582-3p in pan-cancer. The results showed that *miR*-582-3p was significantly highly expressed in a variety of tumors, including lung adenocarcinoma (**Figure 1A**). Further investigation of the TCGA-LUAD dataset showed that *miR*-582-3p expression was significantly higher in lung adenocarcinoma tissues than in adjacent tissues (n = 521, P = 0.018, 95%CI: 0.080-0.873, **Figure 1B**). This finding was further validated *in vitro*. qRT-PCR analysis demonstrated that *miR*-582-3p expression was significantly upregulated in lung adenocarcinoma cell lines A549 and H1299 compared to normal human bronchial epithelial cells BEAS-2B, showing 3-fold (n = 5, 95%CI: 1.678-2.329, P < 0.0001) and 2.6-fold (n = 5, 95%CI: 0.997-2.273, P < 0.001) increases, respectively (**Figure 1C**). Target Scan bioinformatics prediction suggested that the *PTPRCAP* gene contains a binding site complementary to *miR*-582-3p, which indicates that *PTPRCAP* may be the direct target gene of *miR*-582-3p. We performed targeted verification that in the dual luciferase reporter experiment, the experimental group cells transfected with *miR*-582-3p mimics and WT-*PTPRCAP* showed significantly reduced relative luciferase activity compared to the control group transfected with *miR*-NC and WT-*PTPRCAP*; There was no statistically significant difference in relative luciferase activity between the experimental group cells transfected with *miR*-582-3p mimics and MUT-*PTPRCAP* compared to the control group transfected with *miR*-NC and MUT-*PTPRCAP*, indicating that there was indeed targeted binding of *miR*-582-3p to *PTPRCAP* (n = 3, 95%CI:0.189-0.321, P < 0.0001, **Figures 1D, E**). The relative expression levels of *PTPRCAP* mRNA in 18 cases of lung adenocarcinoma and adjacent tissues, as well as normal lung epithelial BEAS-2B cells and lung adenocarcinoma A549 and H1299 cells, were detected by qRT-PCR. The results demonstrated that *PTPRCAP* expression was significantly downregulated in tumor tissues compared with adjacent normal tissues (P = 0.001, **Figure 1F**). Notably, *PTPRCAP* mRNA levels in A549 and H1299 cells showed 70% (n = 4, P < 0.0001) and 50% (n = 4, P < 0.001) reductions respectively relative to BEAS-2B cells(**Figure 1G**). It can be seen that *miR*-582-3p can target and bind *PTPRCAP* to play a role in LUAD.

### The relationship between miR-582-3p and clinicopathological features

**Figure 2:**
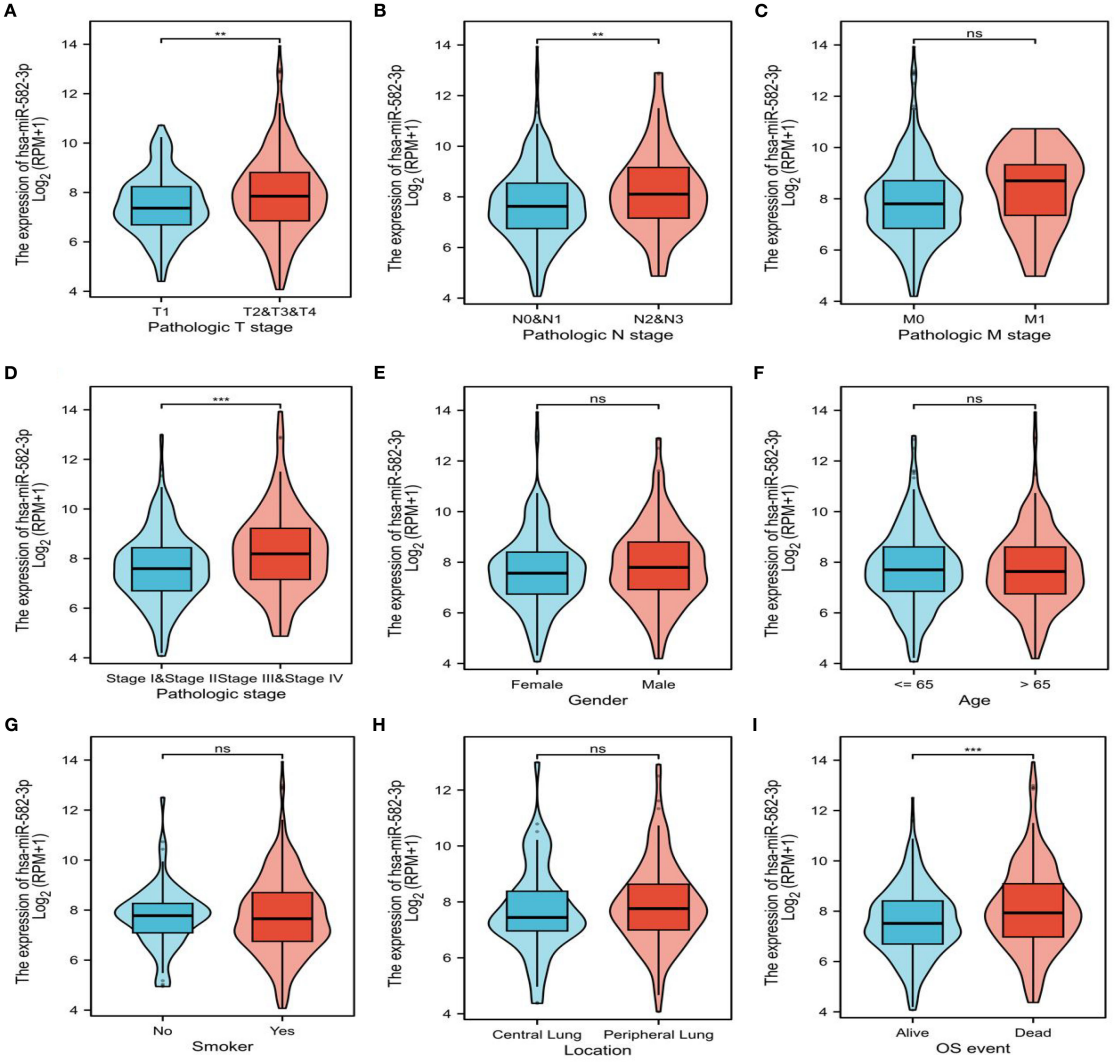
Expression of miR-582-3p in patients with different clinicopathological features and corresponding number of cases. **(A)** T staging (T1 = 178, T2 = 295, T3 & T4 = 68). **(B)** N stage (N0 = 352, N1 = 99, N2N3 = 77). **(C)** M stage (M0 = 370, M1 = 25). **(D)** Pathological stage (stage I = 298, stage II = 127, stage III = 85, stage IV = 26). **(E)** Gender (female = 291, male = 253). **(F)** Age (≤ 65 years = 258, > 65 years = 267). **(G)** Smokers (Yes = 453, No = 77). **(H)** Location (central lung = 64, peripheral lung = 127). **(I)** OS events (survival = 351, death = 193) **P < 0.01, ***P < 0.001.

In this study, we analyzed the relationship between *miR*-582-3p expression and clinicopathological features using *miR*NA-seq data from the TCGA-LUAD (The Cancer Genome Atlas–Lung Adenocarcinoma) dataset, processed according to the BCGSC pipeline. Our results demonstrated that miR-582-3p expression was significantly correlated with T stage, N stage, pathological stage, and overall survival (OS) (**Figures 2A, B, D, I** and **Table 1**), but not with M stage, sex, age, primary tumor location, or smoking status (**Figures 2C, E-H** and **Table 1**). Specifically, *miR*-582-3p expression was significantly elevated in T2, T3, and T4 stage patients compared to those with T1 disease (P = 0.005, 95%CI:0.118-0.626, **Figure 2A**). Similarly, N2 and N3 stage tumors exhibited markedly higher *miR*-582-3p levels than N0 and N1 stage cases (P = 0.006, 95%CI:0.144-0.882, **Figure 2B**). Furthermore, stage III and IV patients showed significantly increased *miR*-582-3p expression relative to stage I and II individuals (P < 0.001, 95%CI:0.296-0.937, **Figure 2D**). Deceased patients exhibited higher miR-582-3p expression than surviving patients (P < 0.001, 95%CI:0.194-0.735, **Figure 2I**). Notably, the high-expression group had a greater proportion of T2-T4 stage disease (36.1% vs. 30.5%, P = 0.010), higher rates of N2-N3 lymph node metastasis (9.3% vs. 5.7%, P = 0.032), and increased prevalence of stage III-IV tumors (13.0% vs. 8.0%, P = 0.006). Importantly, patients with elevated *miR*-582-3p expression exhibited significantly worse mortality (20.9% vs. 14.8%, P = 0.004) (**Table 1**). Cox regression analysis (**Table 2**), adjusted for TNM stage and other confounding factors, confirmed that high *miR*-582-3p expression remained an independent prognostic predictor (HR = 1.440, 95% CI: 1.018-2.037, P = 0.039). The hazard ratio was comparable to those of T2-T4 stage (HR = 1.629, P = 0.030) and stage III-IV disease (HR = 2.376, P = 0.014). Intriguingly, while the N/M stage demonstrated prognostic significance in univariate analysis, it lost statistical significance in the multivariate model. These findings suggest that *miR*-582-3p may influence lung adenocarcinoma progression by enhancing local invasion and lymph node metastasis (**Table 1**). Collectively, our results not only establish *miR*-582-3p as a novel prognostic biomarker but also uncover its biological relevance in key tumor progression pathways, providing a rationale for developing *miR*-582-3p-targeted precision diagnostic and therapeutic strategies.

**Table 1:** Correlation of miR-582-3p expression with patients' clinicopathological features (n, %).

Characteristics	Low expression (n = 260)	High expression (n = 261)	P value
Pathologic T stage			0.010*
T1	100 (19.3)	73 (14.1)	
T2-T4	158 (30.5)	187 (36.1)	
Pathologic N stage			0.032*
N0-N1	222 (43.8)	209 (41.2)	
N2-N3	29 (5.7)	47 (9.3)	
Pathologic M stage			0.463
M0	165 (44.1)	186 (49.7)	
M1	9 (2.4)	14 (3.7)	
Pathologic stage			0.006**
I-II	214 (41.6)	192 (37.4)	
III-IV	41 (8)	67 (13.0)	
Gender			0.271
Female	145 (27.8)	133 (25.5)	
Male	115 (22.1)	128 (24.6)	
Smoking status			0.265
No	33 (6.5)	43 (8.5)	
Yes	217 (42.8)	214 (42.2)	
Tumor location			0.147
Central	36 (19.4)	26 (14)	
Peripheral	58 (31.2)	66 (35.5)	
OS event			0.004**
Alive	183 (35.1%)	152 (29.2)	
Dead	77 (14.8%)	109 (20.9%)	

(*:p<0.05, **: p<0.01).

**Table 2:** Univariate and multivariate Cox regression analysis of prognostic factors.

Characteristics	Total(n)	Univariate analysis		Multivariate analysis	
		Hazard ratio (95% CI)	P value	Hazard ratio (95% CI)	P value
Pathologic T stage	509				
T1	173	Reference		Reference	
T2-T4	336	1.637 (1.165 - 2.300)	0.005**	1.629 (1.049 - 2.532)	0.030*
Pathologic N stage	498				
N0-N1	425	Reference		Reference	
N2-N3	73	2.397 (1.681 - 3.417)	<0.001***	1.143 (0.568 - 2.301)	0.708
Pathologic M stage	365				
M0	342	Reference		Reference	
M1	23	2.330 (1.360 - 3.991)	0.002**	1.025 (0.475 - 2.211)	0.950
Pathologic stage	505				
I-II	400	Reference		Reference	
III- IV	105	2.766 (2.030 - 3.768)	<0.001***	2.376 (1.192 - 4.735)	0.014*
hsa-*miR*-582-3p	512				
Low expression	258	Reference		Reference	
High expression	254	1.382 (1.030 - 1.856)	0.031*	1.440 (1.018 - 2.037)	0.039*

(*: p<0.05, **: p<0.01, ***: p<0.001).

### Relationship between *PTPRCAP* and clinicopathological features and protein expression of PTPRCAP

**Figure 3:**
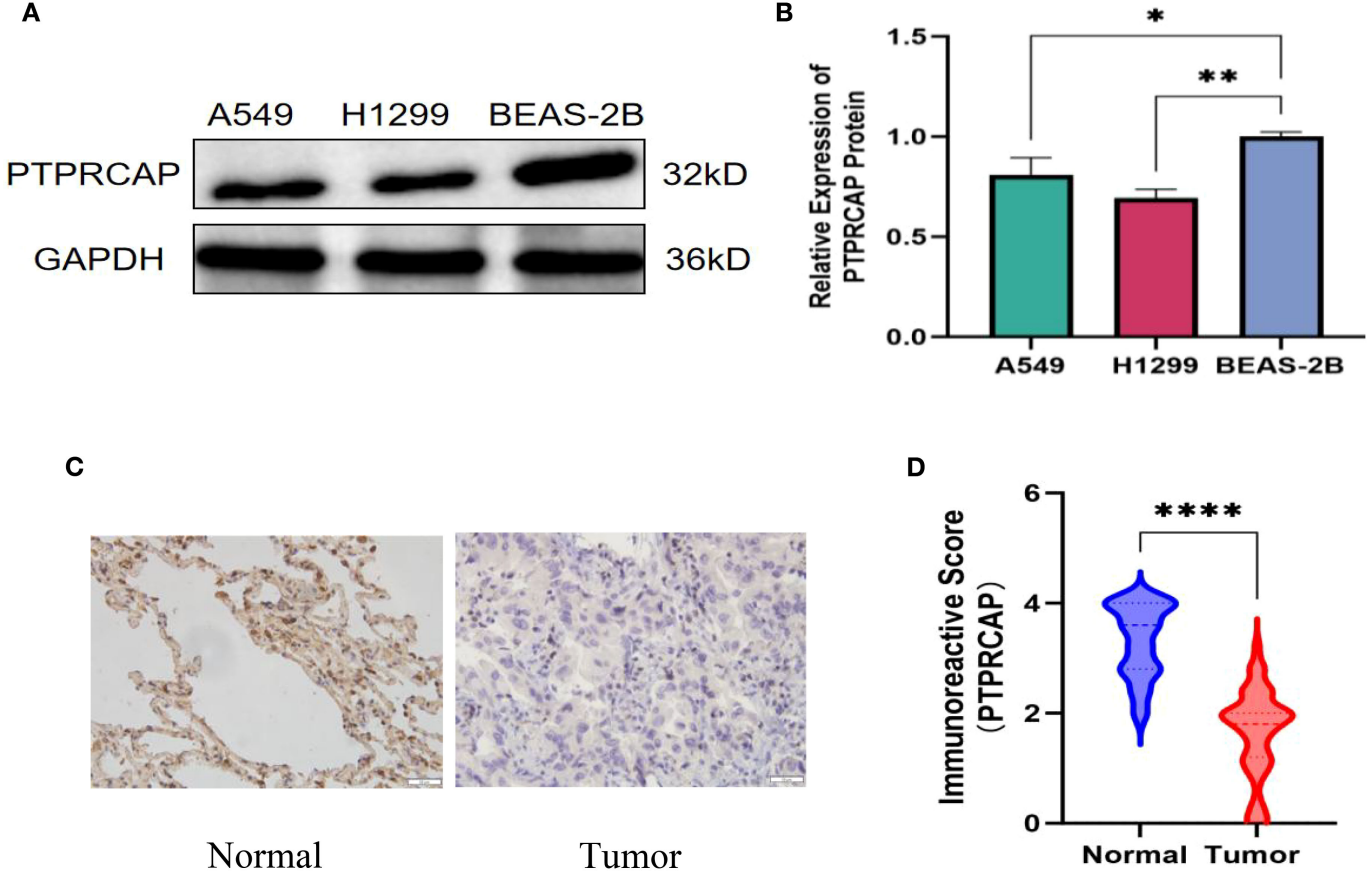
Protein expression of PTPRCAP. **(A)** Protein expression levels of PTPRCAP in BEAS-2B, A549 and H1299. **(B)** Comparison of protein expression of PTPRCAP in BEAS-2B, A549 and H1299. **(C)** Protein expression of PTPRCAP in lung adenocarcinoma cancer tissues and adjacent tissues. **(D)** Comparison of immunohistochemical scores of PTPRCAP protein expression in lung adenocarcinoma and adjacent tissues. *P < 0.05, **P < 0.01, ****P < 0.0001.

The expression of PTPRCAP protein in normal lung epithelial cell line BEAS-2B and lung adenocarcinoma cell lines A549 and H1299 was detected by Western blot, and the expression of PTPRCAP protein in cancer tissues and adjacent tissues of 45 patients with lung adenocarcinoma was determined by immunohistochemistry, and the relationship between the expression level of PTPRCAP protein and clinical features was analyzed. The results of Western blot demonstrated a downregulation of PTPRCAP protein expression in lung adenocarcinoma cell lines A549 and H1299 compared to normal pulmonary epithelial BEAS-2B cells, with reductions of 20% (n = 3, P = 0.015) and 30% (n = 3, P = 0.01), respectively (**Figures 3A, B**). Immunohistochemical analysis of clinical samples revealed that the positive expression rate of PTPRCAP protein in cancerous tissue (22.22%, 10/45) was significantly lower than that in adjacent non-cancerous tissue (93.33%, 42/45), and the IRS score in the cancer group was significantly lower than that in the normal group (median = 1.8 (IQR 1.2–2.0) vs. median = 3.6 (IQR 2.8-4.0); Mann-Whitney U = 101, P < 0.0001, **Figures 3C, D**). There was a statistically significant difference in the positive expression rate of PTPRCAP in patients with different TNM stages and different degrees of tissue differentiation, but there was no statistically significant difference in the positive expression rate of PTPRCAP in patients with different ages, genders, and lymph node metastasis (**Table 3**). The above results indicate that PTPRCAP may play a certain inhibitory role in the occurrence and development of lung adenocarcinoma.

**Table 3:** Correlation of PTPRCAP expression with patients' clinicopathological features (n, %).

Characteristics	Total (n = 45)	PTPRCAP-positive n (%)	χ²	P value
Gender			0.536	0.464
Male	18	5 (27.8)		
Female	27	5 (18.5)		
Age (years)			0.331	0.565
≥60	28	7 (25.0)		
<60	17	3 (17.6)		
TNM stage			7.782	0.005**
I	23	9 (39.1)		
II	22	1 (4.5)		
Differentiation			11.770	0.003**
Well	3	3 (100.0)		
Moderate	39	7 (17.9)		
Poor	3	0 (0.0)		
Lymph node metastasis			0.124	0.725
Positive	6	1 (16.7)		
Negative	39	9 (23.1)		

(**: p<0.01).

### Verification of transfection efficiency of *miR*-582-3p, *PTPRCAP*

**Figure 4:**
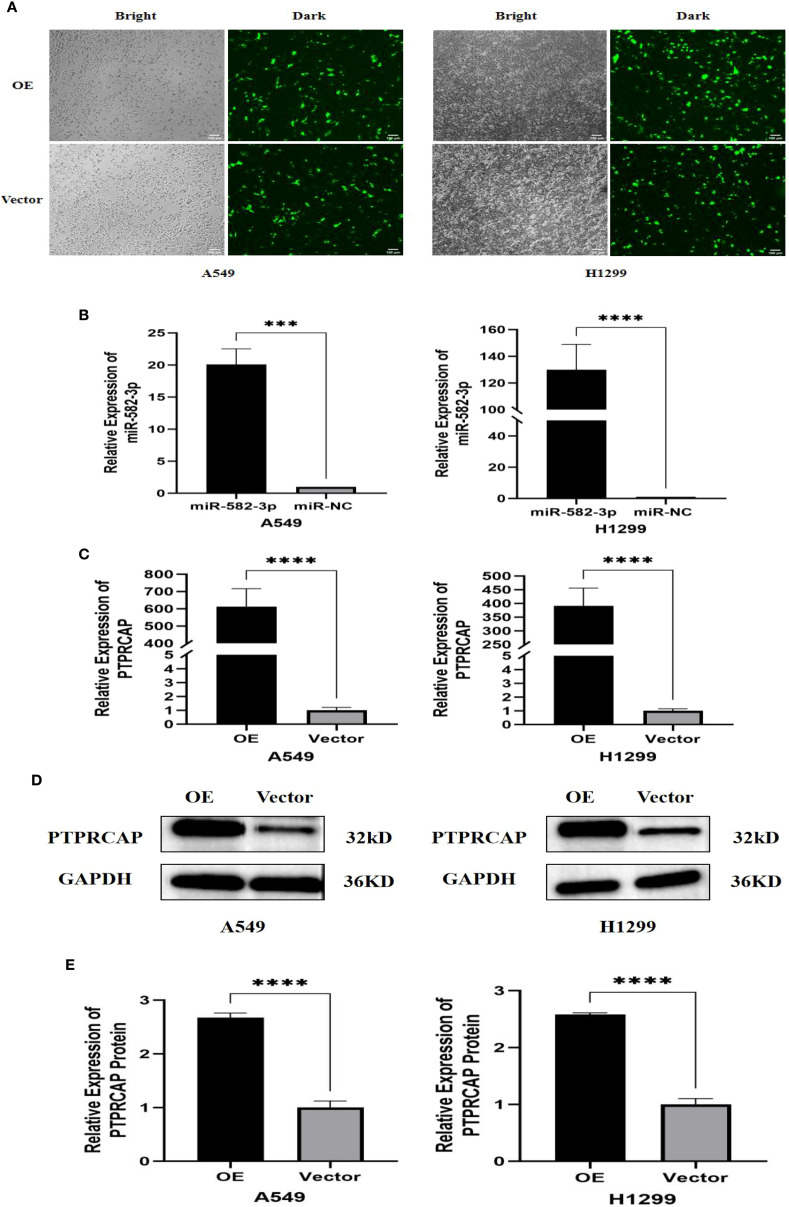
Validation of transfection efficiency of miR-582-3p and *PTPRCAP*. **(A)** Fluorescence transfection of *PTPRCAP* at A549 and H1299. **(B)** Comparison of transfection efficiency of miR-582-3p at A549 and H1299. **(C)** Comparison of transfection efficiency of *PTPRCAP* at A549 and H1299. **(D)** Protein expression levels of each group in A549 and H1299 cells after overexpression of *PTPRCAP*. **(E)** Protein expression comparison of groups in A549 and H1299 cells after overexpression of *PTPRCAP*. ***P < 0.001, ****P < 0.0001.

The control plasmids of *miR*-582-3p mimics, mimics NC, and *PTPRCAP* were transferred into A549 cells and H1299 cells with Lipofectamine 3000 transfection reagent, respectively, and divided into the *miR*-582-3p group, *miR*-NC group, OE group, and Vector group. The transfection efficiency of each group was verified by RT-qPCR and Western blot. After transfection of the *PTPRCAP* overexpression plasmid and the control plasmid, the transfection efficiency was initially revealed under the fluorescence microscope (n = 4, P < 0.001, **Figure 4A**). RT-qPCR results showed that in A549 and H1299 cells, *miR*-582-3p expression was significantly increased following transfection with *miR*-582-3p mimics compared to the negative control (n = 4, P < 0.01, **Figure 4B**). The expression of *PTPRCAP* was significantly higher in the OE group than in the Vector group (n = 4, P < 0.001, **Figure 4C**). This indicates that overexpression models were successfully constructed in lung adenocarcinoma cell lines A549 and H1299. After transfection with the overexpression plasmid, Western blot results showed that the protein expression of *PTPRCAP* in the OE group was significantly higher than that in the Vector group in the two cells (n = 3, P < 0.001, **Figures 4D, E**). Collectively, these findings suggest that each group of overexpression models has been stably constructed in A549 and H1299 cell lines, which can be used for subsequent experiments.

### Effects of upregulated *miR*-582-3p on proliferation, migration, and invasion of lung adenocarcinoma A549 and H1299 cells

**Figure 5:**
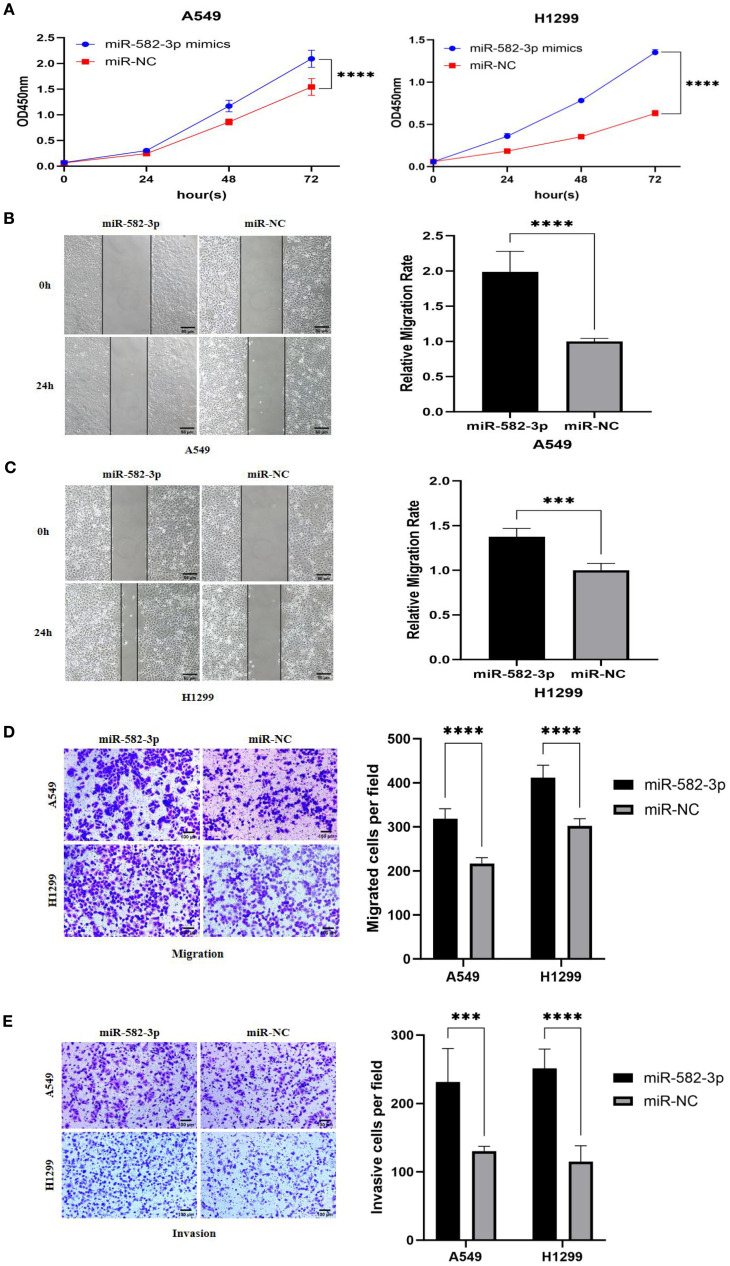
The effects of upregulating miR-582-3p on the proliferation, migration and invasion of lung adenocarcinoma A549 and H1299 cells. **(A)** CCK8 assay to detect the effect of upregulating miR-582-3p on the proliferation ability of A549 and H1299 cells. **(B)** Scratch assay to detect the effect of upregulating miR-582-3p on the migration ability of A549 cells. **(C)** Scratch assay to detect the effect of upregulating miR-582-3p on the migration ability of H1299 cells. **(D)** Transwell chamber migration assay to detect the effect of upregulating miR-582-3p on the migration ability of A549 and H1299 cells. **(E)** Transwell chamber invasion assay to detect the effect of upregulating miR-582-3p on the invasion ability of A549 and H1299 cells. ***P < 0.001, ****P < 0.0001.

Following transfection with *miR*-582-3p mimics or negative control *miR*NA (*miR*-NC), A549 and H1299 cells were allocated into *miR*-582-3p and *miR*-NC groups, respectively. A series of functional assays, including CCK-8 proliferation, wound healing, Transwell migration and invasion, and flow cytometric apoptosis analysis, were conducted to assess the oncogenic effects of *miR*-582-3p. The CCK-8 assay revealed a significant enhancement in proliferative capacity in the *miR*-582-3p group compared to the *miR*-NC group for both A549 and H1299 cells (n = 3, P < 0.001; **Figure 5A**). Concordantly, wound healing assays demonstrated a markedly increased migratory ability in *miR*-582-3p-transfected cells (n = 6, P < 0.001; **Figures 5B, C**). Furthermore, Transwell assays confirmed substantial promotion of both migratory and invasive capacities, as evidenced by increased numbers of migrating and invading cells in the *miR*-582-3p group (n = 5, P < 0.001; **Figures 5D, E**). Collectively, these results demonstrate that *miR*-582-3p functions as an onco*miR* by promoting proliferation, migration, and invasion in lung adenocarcinoma cells.

### Effects of overexpression of *PTPRCAP* on proliferation, migration, and invasion of lung adenocarcinoma A549 and H1299 cells

**Figure 6:**
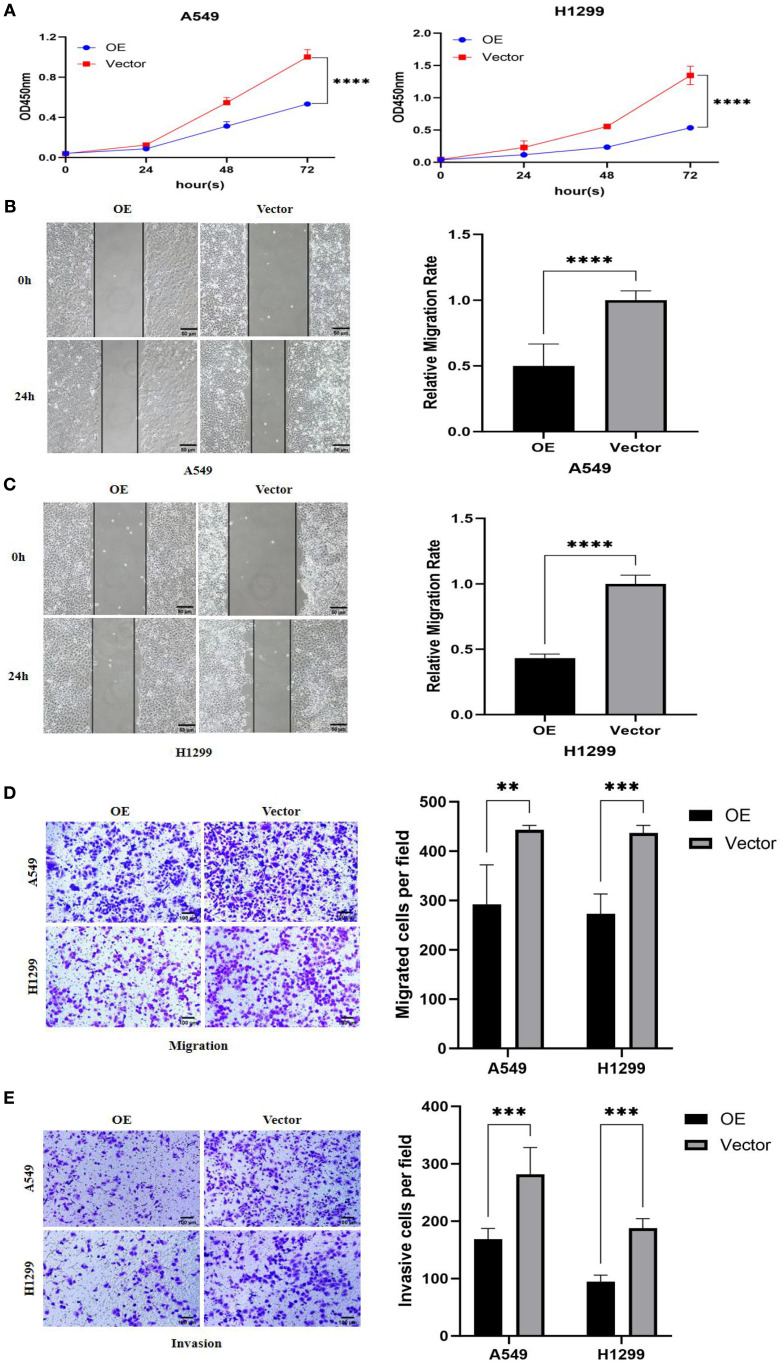
Effects of *PTPRCAP* overexpression on proliferation, migration and invasion of lung adenocarcinoma A549 and H1299 cells. **(A)** CCK8 detects the effect of overexpression of *PTPRCAP* on the proliferation ability of A549 and H1299 cells. **(B)** Scratch assay to detect the effect of overexpression of *PTPRCAP* on the migration ability of A549 cells. **(C)** Scratch assay to detect the effect of overexpression of *PTPRCAP* on the migration ability of H1299 cells. **(D)** The effect of overexpression of *PTPRCAP* on the migration ability of A549 and H1299 cells was examined by Trans well chamber migration assay. **(E)** Trans well chamber invasion assay to detect the effect of overexpression of *PTPRCAP* on the invasion ability of A549 and H1299 cells. **P < 0.01, ***P < 0.001, ****P < 0.0001.

Functional phenotypic assays confirmed the significant tumor-suppressive role of *PTPRCAP* in lung adenocarcinoma cells. Following the establishment of stable *PTPRCAP*-overexpressing cell lines, functional analyses revealed that compared to the empty vector control (Vector) group, *PTPRCAP* overexpression markedly suppressed malignant phenotypes in both A549 and H1299 cells. Specifically, the CCK-8 proliferation assay demonstrated a significant reduction in cell viability after 72 hours, with OD values decreased by 64% and 60% in A549 and H1299 cells, respectively (n = 3; both P < 0.01; **Figure 6A**). Wound healing assays showed that the 24-hour wound closure rate was reduced by 50% and 57% in the two cell lines, respectively (n = 6; both P < 0.001; **Figures 6B, C**). Furthermore, Transwell assays indicated that the number of migrating cells was reduced by 34% and 37%, while the number of invading cells was decreased by 42% and 50%, respectively (n = 4; both P < 0.01; **Figures 6D, E**).

### Effect of upregulating *miR*-582-3p on the expression of *PTPRCAP* protein and Wnt/β-catenin pathway protein

**Figure 7:**
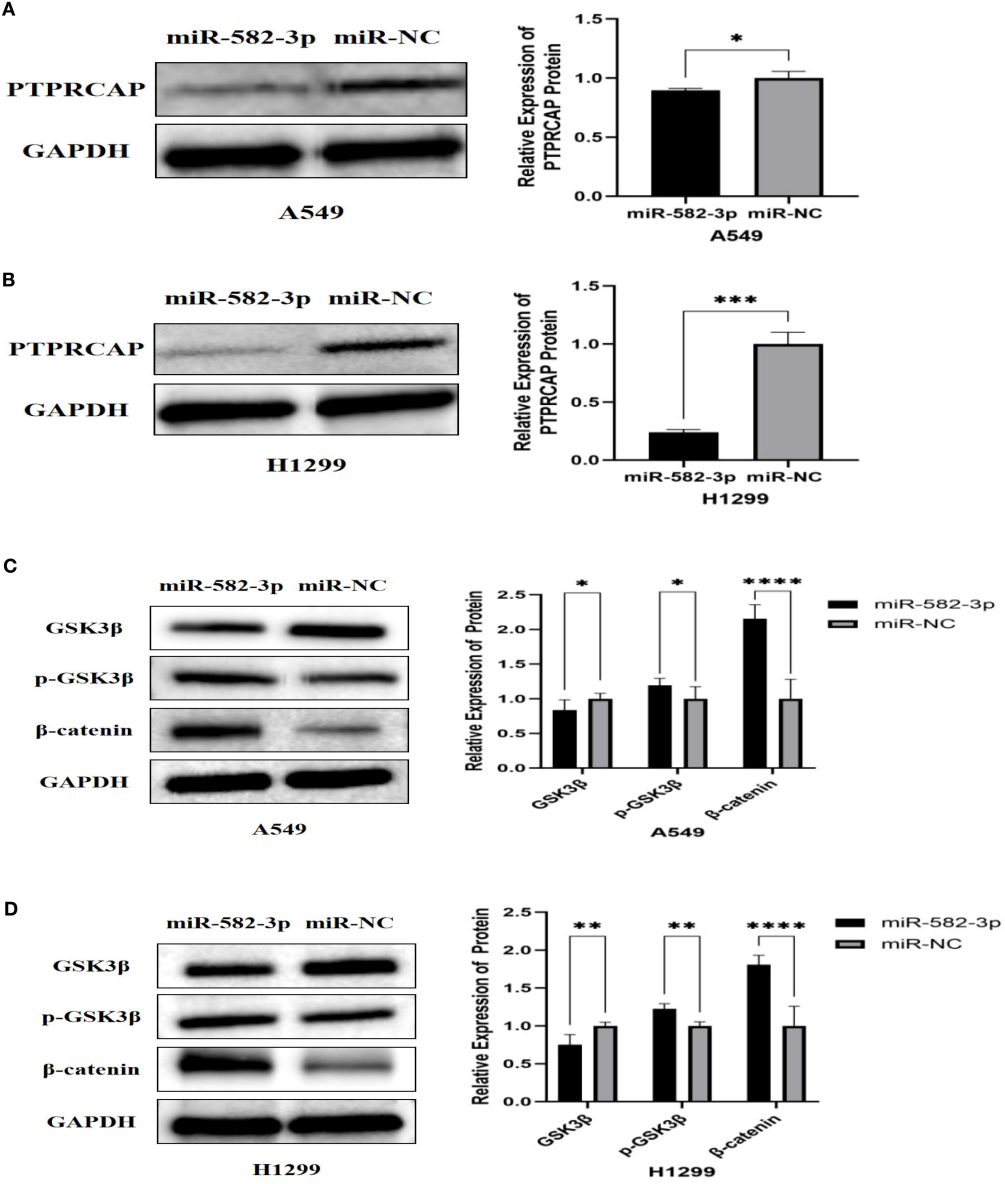
Effect of up-regulation of miR-582-3p on the expression of PTPRCAP protein and Wnt/β-catenin pathway protein. **(A)** Comparison of protein expression levels and relative expression levels of PTPRCAP in A549 after upregulation of miR-582-3p. **(B)** Comparison of protein expression and relative protein expression of PTPRCAP in H1299 after up-regulation of miR-582-3p. **(C)** Comparison of protein expression and relative protein expression levels of GSK3β, p-GSK3β and β-catenin in A549 cells after up-regulation of miR-582-3p. **(D)** Comparison of protein expression levels and relative protein expression levels of GSK3β, p-GSK3β and β-catenin in H129 9 cells after up-regulation of miR-582-3p. *P < 0.05, **P < 0.01, ***P < 0.001, ****P < 0.001.

To investigate the functional impact of *miR*-582-3p upregulation, we assessed the protein expression of PTPRCAP and key components of the Wnt/β-catenin signaling pathway, including GSK3β, p-GSK3β, and β-catenin, by Western blot analysis. Following *miR*-582-3p overexpression, PTPRCAP protein levels were significantly reduced in both lung adenocarcinoma cell lines, A549 and H1299. This downregulation was more pronounced in H1299 cells (80% reduction, n = 3, P < 0.001) than in A549 cells (20% reduction, n = 3, P = 0.030) (**Figures 7A–D**). Furthermore, *miR*-582-3p upregulation led to decreased protein expression of GSK3β, alongside increased levels of its phosphorylated form (p-GSK3β) and β-catenin in both cell lines, with the most notable change observed in β-catenin accumulation (**Figures 7E–H**). Quantitative analysis revealed that in A549 cells, GSK3β expression was reduced by 17% (n = 9, P = 0.004), while p-GSK3β and β-catenin levels were increased by 20% (n = 9, P = 0.011) and 115% (n = 9, P < 0.0001), respectively. Similarly, in H1299 cells, GSK3β expression decreased by 25% (n = 9, P = 0.002), whereas p-GSK3β and β-catenin levels increased by 23% (n = 9, P = 0.005) and 80% (n = 9, P < 0.0001), respectively. Collectively, these results suggest that *miR*-582-3p-mediated regulation of PTPRCAP may potentially function through the activation of the Wnt/β-catenin signaling pathway.

### Effect of overexpression of PTPRCAP on protein expression of the Wnt/β-catenin pathway

**Figure 8:**
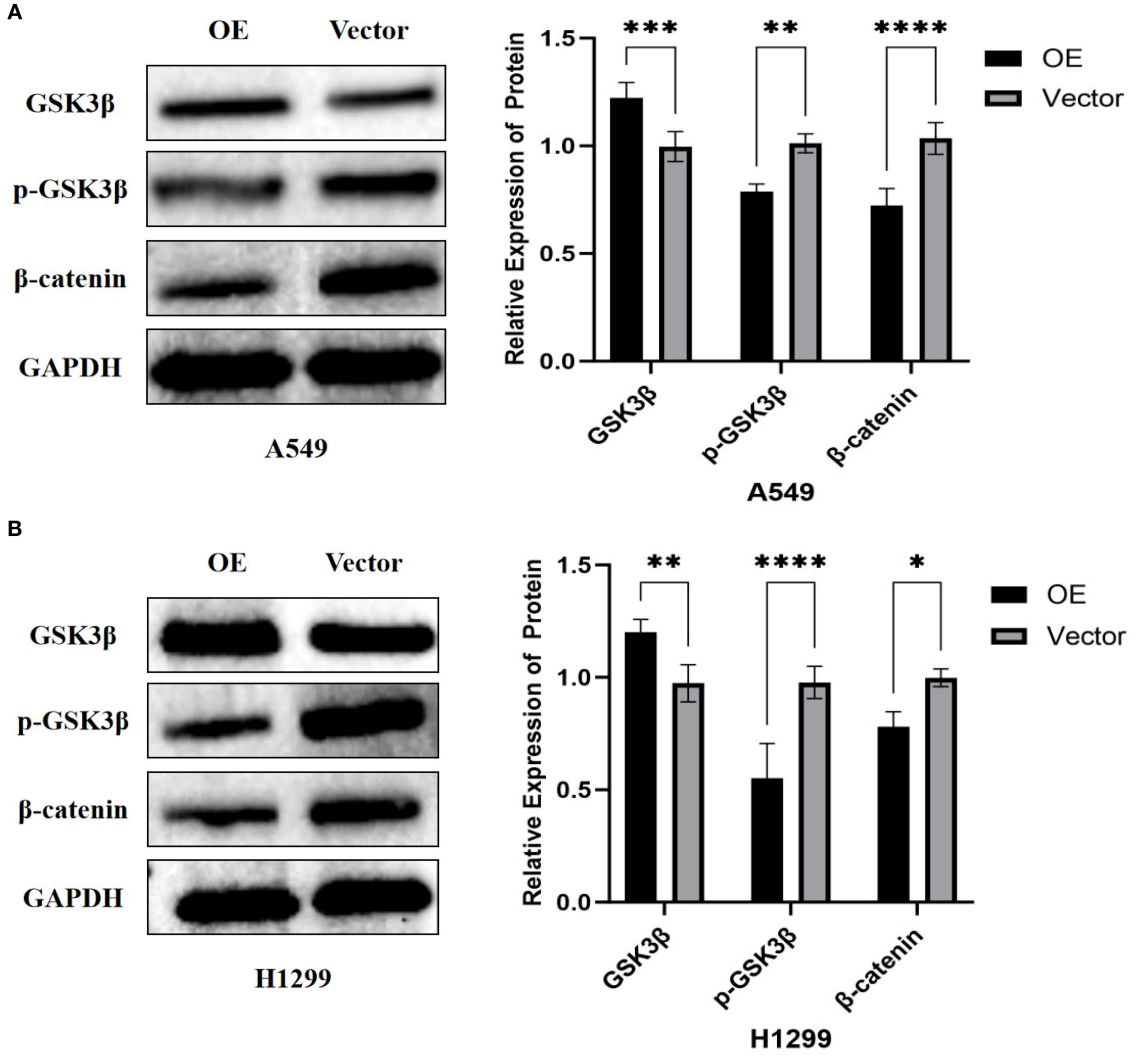
Effects of PTPRCAP overexpression on the protein expression of Wnt/β-catenin pathway components. **(A)** Protein expression levels and quantitative comparison of GSK3β, p-GSK3β, and β-catenin in A549 cells following PTPRCAP overexpression. **(B)** Protein expression levels and quantitative comparison of GSK3β, p-GSK3β, and β-catenin in H1299 cells following PTPRCAP overexpression. *p<0.05. **p<0.01 ***p<0.001 ****p<0.0001.

To investigate the regulatory role of PTPRCAP in the Wnt/β-catenin signaling pathway, we examined the protein expression levels of key pathway components—GSK3β, p-GSK3β, and β-catenin—following PTPRCAP overexpression via Western blot analysis. The results demonstrated that PTPRCAP overexpression in lung adenocarcinoma A549 and H1299 cells significantly increased GSK3β expression while decreasing both p-GSK3β and β-catenin levels (**Figures 8A, B**). Specifically, in A549 cells, GSK3β protein levels were elevated by 23% (n = 3, P = 0.001), whereas p-GSK3β and β-catenin were reduced by 22% (n = 3, P = 0.001) and 31% (n = 3, P < 0.0001), respectively. Similarly, in H1299 cells, GSK3β expression increased by 23% (n = 3, P = 0.008), while p-GSK3β and β-catenin levels decreased by 43% (n = 3, P < 0.0001) and 22% (n = 3, P = 0.011), respectively. These findings suggest that PTPRCAP may suppress the activation of the Wnt/β-catenin pathway by upregulating GSK3β expression, inhibiting its phosphorylation, and consequently promoting β-catenin degradation. This mechanism potentially represents a crucial aspect of PTPRCAP’s tumor-suppressive function.

### Effects of co-transfection of *miR*-582-3p mimics and *PTPRCAP* plasmid on proliferation, migration, and invasion of lung adenocarcinoma A549 and H1299 cells

**Figure 9:**
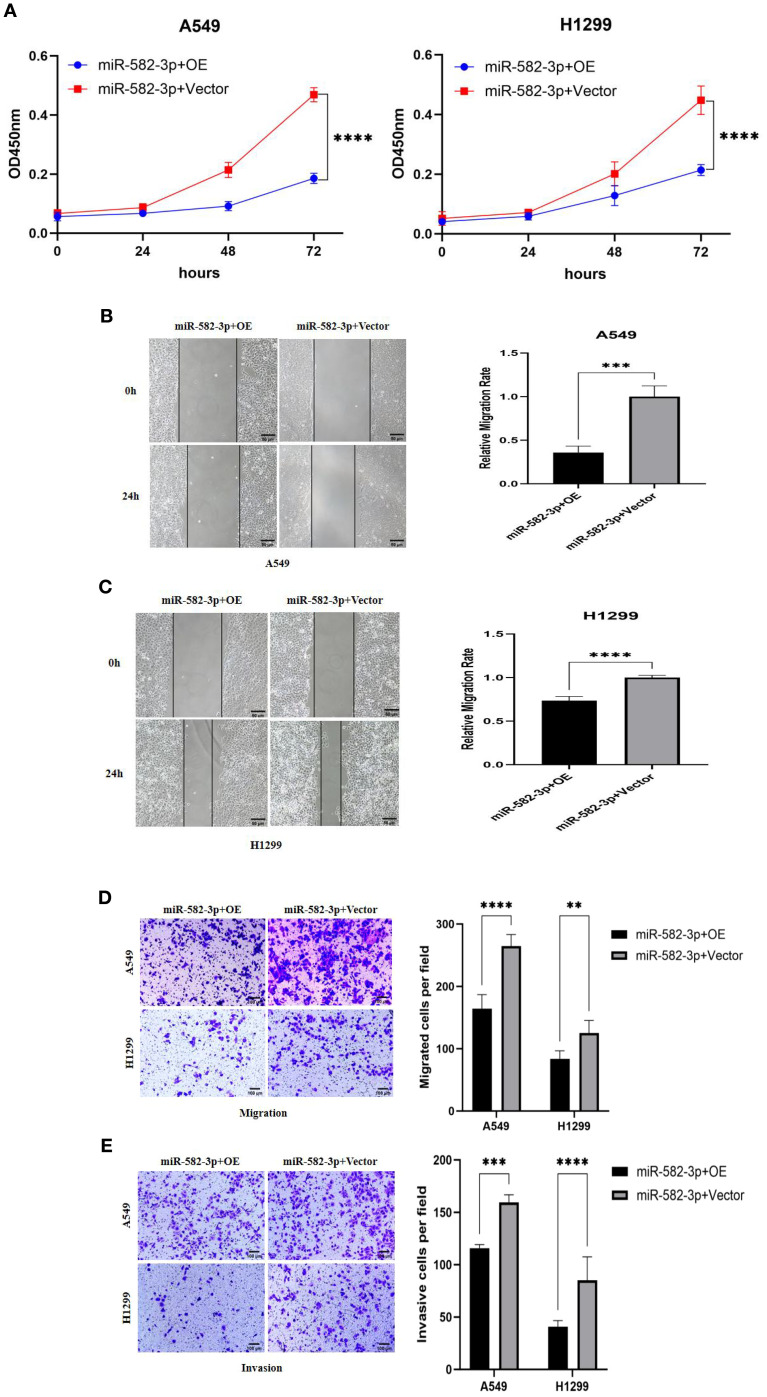
Effects of co-transfection of miR-582-3p and *PTPRCAP* on proliferation, migration and invasion of lung adenocarcinoma A549 and H1299 cells. **(A)** CCK8 assay was used to detect the effect of co-transfection of miR-582-3p and *PTPRCAP* on the proliferation ability of A549 and H1299 cells. **(B)** Scratch assay was used to detect the effect of co-transfection of miR-582-3p and *PTPRCAP* on the migration ability of A549 cells. **(C)** Scratch assay was used to detect the effect of co-transfection of miR-582-3p and *PTPRCAP* on the migration ability of H1299 cells. **(D)** Transwell chamber migration assay was used to detect the effect of co-transfection of miR-582-3p and *PTPRCAP* on the migration ability of A549 and H1299 cells. **(E)** Transwell chamber invasion assay was used to detect the effect of co-transfection of miR-582-3p and *PTPRCAP* on the invasion ability of A549 and H1299 cells. **P < 0.01, ***P < 0.001, ****P < 0.001.

Rescue experiments were performed to investigate whether *PTPRCAP* overexpression could reverse the oncogenic effects of *miR*-582-3p. In A549 and H1299 lung adenocarcinoma cells, co-transfection with *miR*-582-3p mimics and either a *PTPRCAP* overexpression plasmid (*miR*-582-3p+OE group) or an empty vector control (*miR*-582-3p+Vector group) was conducted. Malignant phenotypes were subsequently assessed using CCK-8 proliferation, wound healing, and transwell migration and invasion assays. Compared to the *miR*-582-3p+Vector group, the *miR*-582-3p+OE group exhibited a significant reduction in proliferative capacity in both cell lines (n = 3, P < 0.0001; **Figure 9A**). Consistently, wound healing assays demonstrated markedly impaired migratory ability in the *miR*-582-3p+OE group, with wound closure rates reduced by 64% (A549, n = 4, P = 0.0001) and 30% (H1299, n = 4, P < 0.0001) (**Figure 9B, C**). Furthermore, Transwell assays revealed that the number of migrating cells was decreased by 38% (A549, n = 4, P < 0.0001) and 33% (H1299, n = 4, P = 0.002), while the number of invading cells was reduced by 27% (A549, n = 4, P < 0.001) and 52% (H1299, n = 4, P < 0.0001) in the *miR*-582-3p+OE group (**Figures 9D, E**). These rescue results suggest that the restoration of *PTPRCAP* expression effectively reverses the tumor-promoting phenotypes induced by *miR*-582-3p overexpression, supporting the conclusion that *miR*-582-3p likely promotes malignant progression in lung adenocarcinoma cells, at least in part, through targeted suppression of *PTPRCAP*.

### Effects of co-transfection of *miR*-582-3p mimics and *PTPRCAP* plasmid on the expression of PTPRCAP protein and Wnt/β-catenin pathway protein

**Figure 10:**
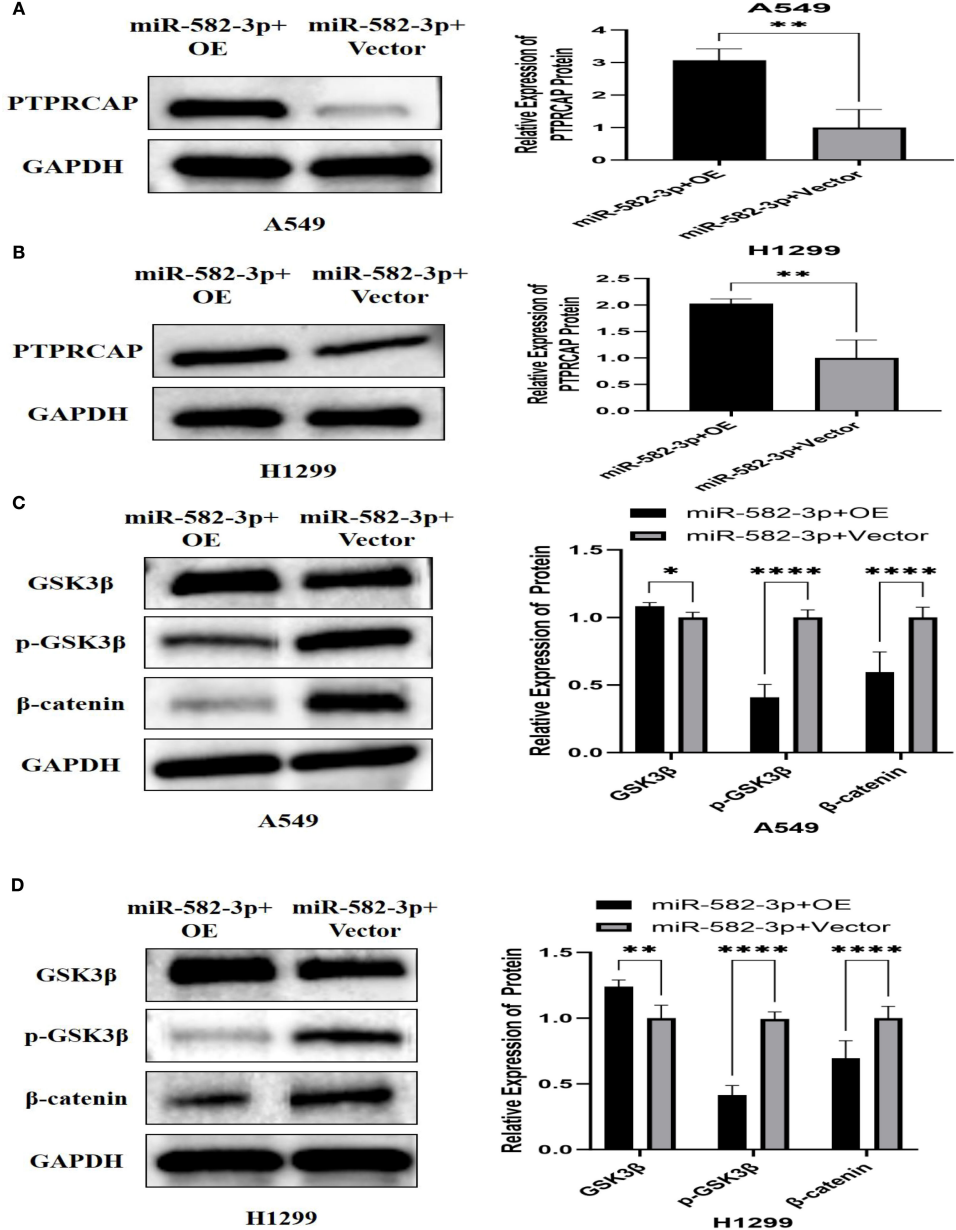
Effect of co-transfection of miR-582-3p mimics and *PTPRCAP* plasmid on the expression of PTPRCAP protein and Wnt/β-catenin pathway protein. **(A)** Comparison of protein expression and relative expression levels of PTPRCAP in A549 after co-transfection of miR-582-3p mimics and *PTPRCAP* plasmids. **(B)** Comparison of protein expression and relative protein expression levels of *PTPRCAP* in H1299 after co-transfection of miR-582-3p mimics and *PTPRCAP* plasmid. **(C)** Comparison of protein expression and relative protein expression levels of GSK3β, p-GSK3β and β-catenin in A549 cells after co-transfection with miR-582-3p mimics and PTPRCA P plasmid. **(D)** Comparison of protein expression and relative protein expression levels of GSK3β, p-GSK3β and β-catenin in H1299 cells after up-regulation of miR-582-3p. *P < 0.05, **P < 0.01, ****P < 0.001.

To investigate the regulatory relationship between *miR*-582-3p and *PTPRCAP* within the Wnt/β-catenin pathway, we co-transfected A549 and H1299 cells with *miR*-582-3p mimics and a *PTPRCAP* overexpression plasmid, followed by Western blot analysis to evaluate expression changes of *PTPRCAP* and key Wnt/β-catenin signaling proteins. Compared to the corresponding control group, the *miR*-582-3p+OE group showed a significant upregulation of PTPRCAP protein expression, with approximately 3-fold and 2-fold increases in A549 (n = 3, P = 0.005) and H1299 cells (n = 3, P = 0.007), respectively (**Figures 10A, B**). Concurrently, this intervention markedly affected the Wnt/β-catenin pathway: GSK3β protein levels increased in both cell lines, while levels of p-GSK3β and β-catenin were significantly reduced (**Figures 10C, D**). Specifically, in A549 cells, GSK3β increased by 10% (n = 3, P = 0.033), p-GSK3β decreased by 60% (n = 9, P < 0.0001), and β-catenin was reduced by 40% (n = 7, P < 0.0001). In H1299 cells, GSK3β rose by 24% (n = 3, P = 0.008), p-GSK3β declined by 58% (n = 9, P < 0.0001), and β-catenin decreased by 31% (n = 9, P < 0.0001). These results provide reverse genetic evidence suggesting that *miR*-582-3p may modulate the activity of the Wnt/β-catenin signaling pathway by targeted suppression of *PTPRCAP*.

## Discussion

Lung adenocarcinoma (LUAD) continues to be a leading cause of cancer-related mortality, with recurrence remaining a significant challenge even in early-stage disease, highlighting an urgent need to decipher its molecular underpinnings for improved therapeutic strategies (23, 24). In this study, we delineate a novel oncogenic pathway in LUAD, wherein *miR*-582-3p promotes tumor progression by directly suppressing *PTPRCAP*, consequently activating the Wnt/β-catenin signaling axis.

MicroRNAs, including *miR*-582-3p, are pivotal post-transcriptional regulators in cancer (25, 26). In nasopharyngeal carcinoma, it suppresses *RAB31* expression by binding to the long non-coding RNA HOXA10-AS, thereby regulating cancer cell proliferation and migration (27). In bladder cancer, it inhibits tumor cell proliferation, migration, and invasion by targeting *KIF3A* (28). In LUAD, Sun et al. demonstrated that *miR*-582-3p directly regulates the expression of the cell cycle-related protein p27, promoting cancer cell proliferation (29). Notably, serum containing Astragalus and Hedyotis diffusa can inhibit A549 cell proliferation via the *miR*-582-3p–p27 pathway, highlighting its potential therapeutic value (29). Prognostic marker studies based on miRNA sequencing data have shown that high expression of *miR*-582-3p is significantly associated with reduced patient survival, indicating its importance as a prognostic indicator (30). Our analysis of the TCGA database revealed that *miR*-582-3p is overexpressed in multiple tumors, including LUAD. Further validation using the TCGA-LUAD dataset confirmed its significantly higher expression in LUAD tissues compared to adjacent non-tumor tissues. Cellular experiments demonstrated that *miR*-582-3p is highly expressed in A549 and H1299 cells, and its upregulation markedly enhanced their proliferative, migratory, and invasive capacities.

Integrating Target Scan prediction with dual-luciferase assays established protein tyrosine phosphatase receptor type C-associated protein (*PTPRCAP/CD45-AP/LPAP*) as a direct target of *miR*-582-3p. PTPRCAP stabilises the phosphatase PTPRC/CD45 (31), yet its role is context-dependent. Early studies showed that the minor allele at the rs869736 locus of the *PTPRCAP* gene promoter enhances promoter activity and nuclear protein binding, upregulating its expression and increasing susceptibility to diffuse gastric cancer; additionally, *PTPRCAP* can promote tumor progression by activating SRC family kinases (SFKs) and disrupting E-cadherin-mediated cell junctions (32). Recent TCGA analyses revealed *PTPRCAP* overexpression in ovarian cancers with DNA damage repair (DDR) deficiencies, where it contributes to a distinct immune signature (33). In breast cancer, MARCHETTI et al. combined bioinformatics with RT-qPCR and Western blot analyses to demonstrate that *PTPRCAP* expression is positively correlated with disease-free survival in triple-negative breast cancer patients, while its expression is low in corresponding cell lines (34). Proteogenomic profiling indicates that LUAD *PTPRCAP* abundance is controlled by DNA methylation (35), and stemness-index analyses uniquely associate *PTPRCAP* with stemness signatures in both blood and tumour tissue (36). We confirmed markedly reduced PTPRCAP mRNA and protein in 18 LUAD specimens and in A549 and H1299 cells; immunohistochemistry in 45 paired samples showed positivity in only 22% of tumours versus 93% of adjacent normal lung. Functional rescue demonstrated that *PTPRCAP* re-expression suppressed proliferation, migration, and invasion, confirming its tumour-suppressive role in LUAD.

We further elucidated the connection between this axis and the canonical Wnt/β-catenin pathway—a well-established driver of oncogenesis (37, 38) that contributes critically to breast (39), gastric (40), pancreatic (41), and colorectal carcinogenesis (42). In neuroblastoma, circ_0000285 sponges *miR*-582-3p, relieving its inhibition of GSK-3β, activating β-catenin, and thereby promoting the Wnt/β-catenin pathway and tumor progression (43). Conversely, in hepatocellular carcinoma, XU et al. found that miR-582-3p targets *RRM2* to prevent GSK-3β dephosphorylation, block β-catenin nuclear translocation and subsequent c-Myc activation, ultimately inhibiting Wnt/β-catenin signaling and tumor progression (44). In lung cancer, Wnt/β-catenin pathway activation reduces GSK3β levels while increasing phosphorylated GSK3β (p-GSK3β, Ser9) levels, leading to β-catenin stabilization and accumulation, thereby enhancing cell proliferation, invasion, and metastatic potential (45, 46). Our data demonstrate that *miR*-582-3p upregulation or *PTPRCAP* knockdown activates the pathway, increasing levels of p-GSK3β (Ser9) and active β-catenin. Conversely, *PTPRCAP* overexpression had the opposite effect. This finding provides a crucial mechanistic bridge to the work of Fang et al. (22), who showed that *miR*-582-3p activates Wnt/β-catenin signaling to maintain stem-like properties; we propose that *PTPRCAP* is the functional target mediating this activation. Furthermore, the predicted involvement of *PTPRCAP* in Wnt signaling (47) and the association of its binding partner *PTPRC* with poor survival in NSCLC (48) lend further support to our model.

Despite these insights, our study has limitations. First, the clinical sample size for validation was limited; larger multi-center cohorts are needed to firmly establish the prognostic value of the *miR*-582-3p/*PTPRCAP* signature. Second, our mechanistic conclusions are primarily based on gain-of-function experiments; future studies employing knockdown/knockout models, especially *in vivo*, are essential. Third, the direct molecular mechanism connecting PTPRCAP to the regulation of GSK3β phosphorylation remains to be fully uncovered, warranting further investigation through co-IP and phosphoproteomics.

In summary, our data indicate that both *miR*-582-3p and *PTPRCAP* are involved in the pathogenesis of LUAD. We provide evidence that *PTPRCAP*, as a direct target of *miR*-582-3p, mediates its oncogenic effects, at least in part, by negatively regulating Wnt/β-catenin signaling, thereby controlling the proliferation, migration, and invasion of LUAD cells.

Our findings contribute to the growing body of literature on context-dependent miRNA–PTP interactions in cancer. The *miR*-582-3p/*PTPRCAP* axis adds a new layer to this complex regulatory network. Furthermore, our work aligns with and expands upon the study by FANG et al. (22), who showed *miR*-582-3p activates Wnt/β-catenin in lung cancer stem cells; we propose *PTPRCAP* as a novel and critical mechanistic link mediating this activation.

While these findings illuminate a potential new regulatory node in LUAD, the path to therapeutic application is long and fraught with challenges. The development of *miR*-582-3p antagonists (e.g., antagomiRs) or strategies to restore *PTPRCAP* function represents a compelling but speculative future direction. The significant hurdles of *in vivo* delivery, off-target effects, and the context-dependent functions of both the miRNA and its target gene must be thoroughly addressed in pre-clinical models. Therefore, we posit that the primary immediate value of our work lies in enhancing the mechanistic understanding of LUAD progression and offering a potential biomarker signature (*miR*-582-3p high/*PTPRCAP* low). Whether this axis can be therapeutically harnessed remains an open question for extensive future investigation.

## Conclusion

Our findings establish a novel regulatory axis in lung adenocarcinoma (LUAD) pathogenesis, in which *miR*-582-3p directly targets *PTPRCAP* and represses its expression. This *miR*-582-3p/*PTPRCAP* interaction promotes malignant phenotypes in LUAD cells—including proliferation, migration, and invasion—through activation of the Wnt/β-catenin signaling pathway. These results reveal a previously undescribed mechanism contributing to LUAD progression and suggest that both *miR*-582-3p and *PTPRCAP* may represent potential biomarkers for this malignancy.

## Data Availability

The original contributions presented in the study are included in the article/supplementary material. Further inquiries can be directed to the corresponding author.
